# Anti-bribery and Corruption Policies in International Sports Governing Bodies

**DOI:** 10.3389/fspor.2021.649889

**Published:** 2021-05-17

**Authors:** Christina Philippou, Tony Hines

**Affiliations:** Accounting and Financial Management, University of Portsmouth, Portsmouth, United Kingdom

**Keywords:** bribery, corruption, governance, sport, internal controls

## Abstract

International Sports Governing Bodies (“ISGBs”) are diverse in their aims but share a need to maintain a reputation of accountability in the eyes of their stakeholders. While some literature analyses the general governance concerns faced by these organizations, there is limited focus on anti-bribery and corruption (“ABC”) within this sphere. This paper's research aim is an exploratory evaluation of the ABC best practice policies that exist within ISGBs, asking how they can be assessed and what best practice policies currently exist within this framework. This paper undertakes a critical review of the diverse ABC governance policies in the largest ISGBs through content analysis on governance documents publically available on the sample ISGB websites. This review was undertaken twice on the same ISGBs, in 2017 and 2020, and the changes reviewed. The research highlights best practice policies for recommendation to all ISGBs, and illuminates the absence of adequate policies with regards to the risk of bribery in ISGBs. The findings show there was no area within the framework that ISGBs performed well at as a collective, and there was no single ISGB whose anti-bribery policies were strong in all areas. However, the comparison between 2017 and 2020 shows an improvement in ABC policies in some ISGBs over the timeframe analyzed. The implications are a need for sharing best practice in this area of governance, and providing global guidance on ABC policies for ISGBs to ensure integrity in the sector.

## Introduction

Bribery in sport is not an uncommon phenomenon. From boxer Eupolos bribing fellow Olympic Games competitors in 388 BC (Spivey, 2012), to bookmakers bribing stable-boys to dope horses in the 1960s (Reid, 2014), to FIFA Executive Committee members being bribed to secure their votes (Blake and Calvert, 2015; Conn, 2018), sport is awash with examples of this form of corruption.

Bribery and corruption in international sport are rife, systemic, widespread, and linked to stakeholders from athletes to sponsors, although the governance side of the game has gained most attention. For example, FIFA's 2015 governance corruption scandal led to most of their Executive Committee indicted in the US or sanctioned internally (Conn, 2018).

Bribery damages the integrity and image of sport (Gorse and Chadwick, 2010; Kihl et al., 2017) and undermines efficiency and growth within the industry (Azfar et al., 2001). Despite continuing vulnerability to this form of corruption, there is limited literature on how International Sports Governing Bodies (“ISGBs”) tackle bribery through the use of anti-bribery and corruption (“ABC”) corporate governance and ethics policies.

There are also no global, functional best practice guidelines available for implementation by sport organizations (Michie and Oughton, 2005), although some countries have created their own, linked to public funding availability (Australian Sports Commission, 2015, 2020; Sport England UK Sport, 2016). The UN (2017) has also issued guidance on ABC measures for countries tackling corruption in sport. ABC policies are required to ensure that ISGBs can be held to account, and clear policies allow for sanctions against breaches. Absence of adequate policies therefore affects propensity for corruption, although benchmarking of individual ISGB's ABC governance is outside the scope of this paper.

Challenges faced by the sport industry in designing and implementing ABC policies include different structures and hierarchies within ISGBs (Chappelet and Mrkonjic, 2013; Pielke, 2016; Gardiner et al., 2017), lack of awareness of governance problems leading to conflict of interest and fraud (Brooks et al., 2013; Kirkeby, 2016), and ability to indulge in regulatory arbitrage for country of incorporation (Geeraert et al., 2014; Pielke, 2016). For example, the 2015 FIFA scandal was linked to problems with structure of both ISGB and member federations (Tighe and Rowan, 2020), conflicts of interest (Blake and Calvert, 2015), and the protection that Swiss company law previously afforded ISGBs (Associated Press, 2014).

As ISGBs are the regulators of their sport, an evaluation of their ABC policies is required to understand the problem, and provide best policy recommendations to other ISGBs. This paper's research aim is an exploratory qualitative evaluation of ABC policies of ISGBs with regards to policy content and language. This is done against the anti-bribery framework developed by Philippou (2019) for assessing ABC policies based on interdisciplinary corruption research. The intention is to highlight best practice policies (and those missing) within this framework currently adopted by some ISGBs, and outline issues raised on the risk of bribery in ISGBs as a group.

This paper's contribution to knowledge is a critical review of the diverse current ISGB anti-corruption governance policies for the prevention of bribery.

The next section of this paper argues that corporate governance policies are applicable to ISGBs, then provides an overview of ABC literature, followed by a section that outlines the framework used and method employed in the assessment of ABC policies, and a discussion of the results by framework element.

## Corporate Governance and Sport

The European Sports Charter states that “voluntary sports organizations have the right to establish autonomous decision-making processes within the law” (Council of Europe, 2001, Article 3.3). While autonomy has led to self-regulation (Forster and Pope, 2004; Forster, 2006; Chappelet, 2016), some researchers have argued that ISGBs are indeed corporations despite this status (Szymanski and Kuypers, 2000; Barker, 2013). Smith and Stewart (2010) noted that the unique features of the sport industry have diminished since the 1990s from ten (Stewart and Smith, 1999) to four, including having legally allowable monopolistic and/or oligopolistic structures, supporting corporate governance policy applicability to sports organizations.

Governance provides solutions to issues identified by agency theory (Jensen and Meckling, 1976) as applied to sport. Agency problems can be caused by separation (Berle and Means, 1930) between principals (resource allocators and stakeholders such as fans and athletes) and agents (managers of these resources, such as ISGBs).

ISGBs have developed into large revenue-takers and increased their visibility (PWC, 2011, 2016; Gardiner et al., 2017).

Corporate governance of ISGBs is thus increasingly important to governments and policy-makers. Political bodies such as the Council of Europe now regard sport governance as a key issue; they approved the 2013 Berlin Declaration calling for the sport industry's engagement with corporate governance issues (Geeraert, 2016; Gardiner et al., 2017) and adopted the Good Football Governance Resolution (Council of Europe, 2018).

Given the autonomy principle, with sport given special dispensation under law (Council of Europe, 2001), it should be unsurprising that ISGBs are different in their governance and board structure when compared to other corporate organizations, particularly with regards to lack of accountability,. This is especially so when those charged with governance are uninterested, unaware, and/or unable to recognize corruption (Brooks et al., 2013; Kirkeby, 2016).

Proposed solutions to corporate governance problems (and links to corruption) faced by ISGBs put forward by researchers and policy-makers include:

- benchmarking (Geeraert, 2016) and reporting on corporate governance measures (Chappelet and Mrkonjic, 2013);
- accountability for members' actions, including controls over receipt and use of funds (Ionescu, 2015; Pielke, 2016);
- improving transparency, including disclosure of senior management salaries, and procurement methods (Geeraert et al., 2013; Maennig, 2016; Menary, 2016; Transparency International, 2016); and
- providing examples of good governance for other sports governing bodies to follow (Pedersen, 2016) through a best practice code (Michie and Oughton, 2005; Pielke, 2016).

Researchers have attempted to develop benchmarking tools for assessing the strength of corporate governance structures in sport organizations (not necessarily ISGBs). However, if autonomy and self-regulation are indeed part of the reason for poor governance across the sporting industry, then comparisons with peer organizations would be of limited value as an ABC tool. The *Action for Good Governance in International Sport*'s (“AGGIS”) benchmarking tool targeted the areas of transparency, and checks and balances. Both transparency and accountability linked to checks and balances are frequently used controls in the ABC sphere (Solomon, 2013, pp. 151–190) and are covered later in this paper.

Chappelet and Mrkonjic (2013) composed a set of indicators for measuring corruption in sports governing bodies, including organizational and reporting transparency, control mechanisms, and sport integrity, which overlap with the ABC framework (Philippou, 2019) used in this paper. Other benchmarking that has been applied to sport governance includes Play The Game's National Sports Governance Observer (Geeraert, 2018; Alm, 2019). There was, however, no explicit coverage of anti-bribery measures within the benchmarking assessments, and this is a suggested area for further research.

Limited research exists on ABC elements within sport governance. One example includes Pielke (2016), who assessed the conflict of interest and other ABC measures at FIFA against a framework of accountability mechanisms (including legal, market, peer, and public reputational accountability), but not stakeholder accountability. Over the same period, FIFA did well in the AGGIS benchmarking, coming second in the list of 35 Olympic sports federations (Geeraert, 2015).

The methods noted above have been rarely adopted with an emphasis on ABC, although attempts to increase transparency across a number of organizations has taken place over time, and there is limited research into ABC corporate governance applications for ISGBs. This paper aims to begin the process of addressing this paucity of knowledge by analyzing best practice as a first step toward an ABC best practice code in line with Michie and Oughton (2005) and Pielke (2016).

## Bribery

Like corruption (Ashforth and Anand, 2003; Den Nieuwenboer and Kaptein, 2008; Gorse and Chadwick, 2010; Rose, 2017), bribery encompasses an array of definitional issues and is affected by public sector literature bias. This may be in part due to the sense in which sport is a public good even if the bodies running it are not.

ISGBs are, usually, privately incorporated associations, in which corruption is often internal to organizations (vote-rigging, fraud, match-fixing), although senior executives have held public office alongside their ISGB roles. One notable exception is the hosting, by countries, of major sporting events such as the Olympics and the FIFA World Cup. For these events, government-provided infrastructure and entertaining of ISGB members by public officials is often required, and external bribes and procurement fraud may occur (Dorsey, 2015).

Bribery can be defined as the “offering, promising, giving, accepting or soliciting of an advantage as an inducement for an action which is illegal, unethical or a breach of trust” (Transparency International, 2017b). This definition is broader than public-sector definitions (such as that of the Foreign Corrupt Practices Act (Sarbanes-Oxley, 2002) in the US), despite raising perspective issues through not defining the terms “ethics” and “breach of trust,” and is therefore the one used in this paper.

There are limited empirical studies on bribery. Hanousek and Kochanova (2016) found “local bribery environments” affected firm performance in European countries, although the focus was on public-sector officials. Rodrigues-Neto (2014) modeled different forms of bribery to show that where monetary bribes are paid, bargaining power of bribe-payers is relatively small. Other works focus on detection or bribery within the framework of corruption (see, for example, Ryvkin et al., 2017) or on problems associated with bribery from a business perspective (Bray, 2007; Transparency International, 2011). These latter studies are based on perception, measuring beliefs rather than quantity (Sampford, 2006; Brooks et al., 2013). This paper analyses ABC policies rather than quality or quantity of bribery incidences, although perception does play a part in reputational damage suffered by companies as a result of corruption.

## Theoretical Framework

There is a limited range of theoretical frameworks available for critical evaluation of ABC policies. One such example is De Waegeneer et al. (2016), who created a classification framework for content analysis of ISGBs' ethical codes' effectiveness. This included thematic and procedural classifications of content, both of which are relevant to general governance policies, but not explicitly concerned with ISGBs. Another is the TASP sport corruption typology of Masters (2015), which can be applied explicitly to instances of corruption in sport or framing specific scandals within ISGBs.

Svensson (2005) describes corruption as an outcome ‘of a country's legal, economic, cultural and political institutions'. Bribery, in turn, is an outcome of a number of similar variables, both thematic and procedural, which need to be addressed in an ABC policy.

**Figure 1:**
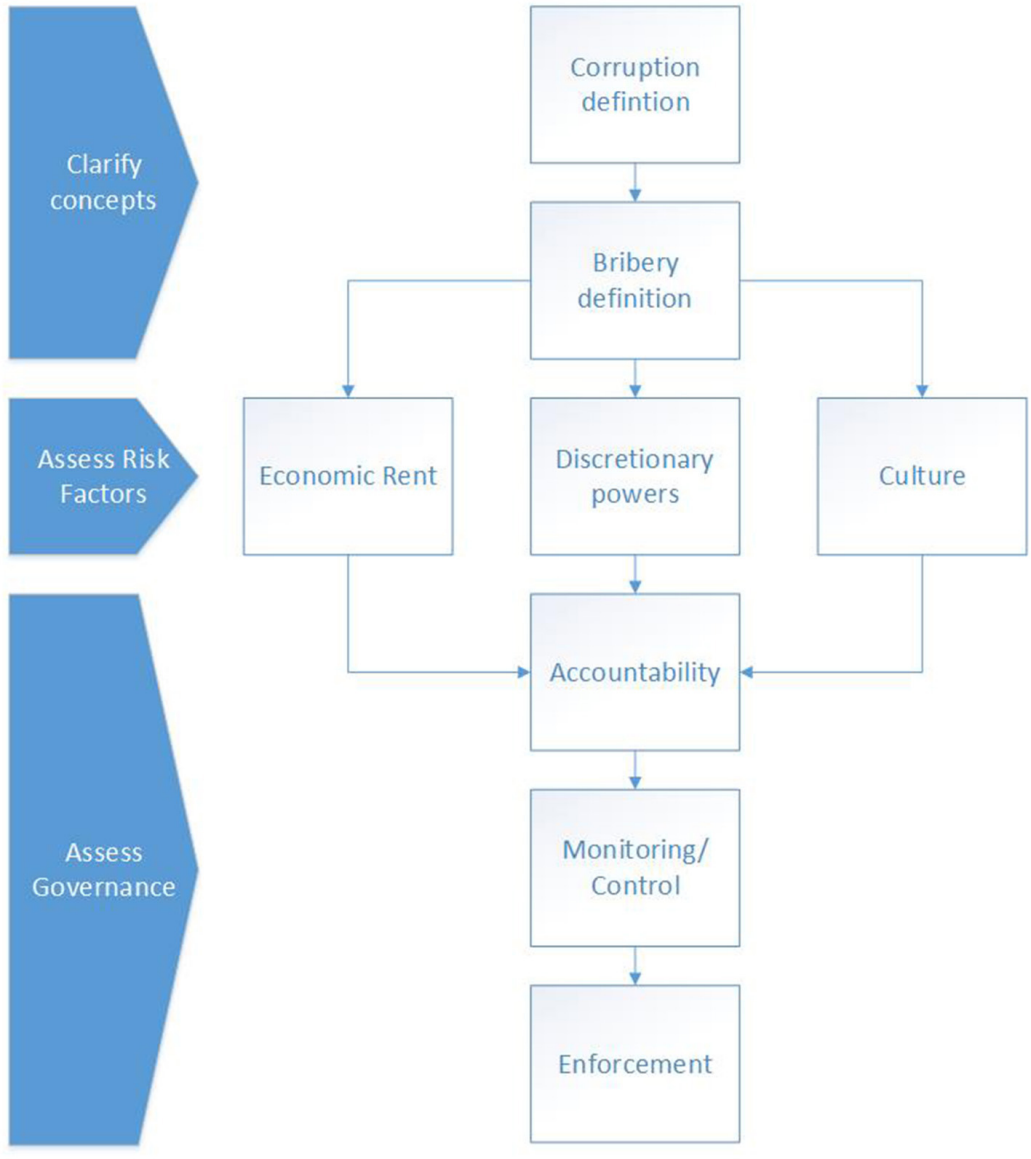
Theoretical framework for bribery and ABC. Adapted from Philippou (2019).

Philippou (2019) sets out a theoretical framework for bribery in sport governance. The framework (Figure 1) is split into three parts: clarifying concepts (such as definitions of corruption and bribery employed), assessing risk factors (economic rent, discretionary powers, and culture), and assessing governance (accountability, monitoring/control systems, and enforcement). As this framework is explicitly concerned with ABC in sport governance, and its production based on an amalgamation of interdisciplinary ABC research, this is the framework used in this paper. Its elements and the relevant literature are discussed below.

## Clarifying Concepts: Definitions

The global scope and activities of ISGBs makes them subject to varied ABC legislation and regulations, which internal policies and codes need to comply with. The ISGBs' ability to take advantage of regulatory arbitrage (such as the ICC's move from the UK to Dubai in 2005) affects the policies and procedures required and therefore enacted.

The US FCPA's (1977, §78dd-3) definition of bribery requires involvement of a public official (UK Government, 2010). The UK Bribery Act 2010 has a much broader definition of bribery, covering private sector bribery (and therefore ISGB officials), passive as well as active bribery (both giving and receiving a bribe), and facilitating payments. Facilitating payments are inducements given to officials to perform routine business transactions in their (legal) job. Facilitating payments are allowable under the FCPA (Baughn et al., 2010), although enforcement has tightened up in recent years.

ISGBs could potentially be affected by public-sector facilitating payment definitions during sport event management and related procurement activities, or during broadcast rights negotiations. ISGBs could also be affected by private-sector bribery in, for example, requesting support in the form of votes in exchange for allocating funds to specific development programs.

Given the reputational risk from being embroiled in a bribery scandal (Gorse and Chadwick, 2010), ISGBs should also include unactioned (agreed but not acted upon) bribery in their definitions. This is because mere agreement to conduct bribery could still damage the ISGB's reputation, as is the case with the unproven allegations of match-fixing in tennis (Mitchell, 2016; see, for example, Blake, 2016). By extension, when these practices become endemic to the culture, it's woven into the fabric of the sport, as was the case with the cultural problems experienced by the Australian cricket team (Lemon, 2018).

## Assessing Risk Factors

### Economic Rent

Policy-driven corruption theory is steeped in the tradition of Klitgaard's (1988) formula and Rose-Ackerman's (1999) framework, both of which attempt to understand (and reverse) the causes of corruption. Both are limited to public-sector corruption [although Klitgaard (1988, 1998b) does acknowledge the existence of private-sector bribery]. As bribery is a subset of corruption, both include economic rent in their respective theoretical frameworks affecting ABC.

Economic rent is the concept of monopoly profit; it is an unsustainable pricing level in the presence of competition (Ricardo, 1821; Krueger, 1974). Alberto Ades and Rafael Di Tella (1999) found that countries whose firms benefit from higher levels of economic rent are more prone to corruption. Clarke and Xu's (2004) regression analysis of bribery in the utility sector in transition economies also found economic rents to be a corrupting factor, and bribery more likely in areas with lower levels of competition and higher profitability.

ISGBs, by their very nature as global organizations, have monopoly power over their sport or (in the case of the IOC) event (Morgan, 2002). An exception to this is the oligopolistic structure of professional [as opposed to amateur and professional, which was governed by the AIBA (2018), despite their authority over Olympic events being rescinded by the IOC (IOC, 2019; Morgan, 2020)] boxing governance, which includes four main ISGBs (the World Boxing Association, World Boxing Council, IBF/USBA, and the World Boxing Organization). However, this is still sufficiently limited to allow the ISGBs to extract economic rents from fans and other stakeholders of the sport.

### Discretionary Powers

Discretionary powers of governance officials affect levels of corruption (Klitgaard, 1988; Rose-Ackerman, 1999; Jain, 2001). Autonomy enjoyed by sport governing bodies under law (Geeraert et al., 2014, 2015; Chappelet, 2016) increase the levels of discretionary powers that governance officials have over their sport.

### Culture

Clearly defining bribery affects behavior (Steidlmeier, 1999; Transparency International, 2013a, Article 5.6.1). Cultural attitudes to bribes affect tendency to both pay (Pitt and Abratt, 1986) and receive (Lambsdorff and Frank, 2010) bribes. Thus, care must be taken where “there are deep-rooted customs regarding gifts and hospitality” (Transparency International, 2017a, Article 6.7), as well as other risk areas, as a result of the global coverage of ISGBs.

Gifts and entertainment (or hospitality) is an important area of ABC (Transparency International, 2013a, 2017a), and forms part of cultural control. The need for gifts and entertainment in ISGBs should be assessed as part of risk. There is also a need to provide guidance on appropriate (sometimes zero) levels above which receipts or donations could be construed as bribery. For example, the UK Bribery Act 2010 [in contrast to the US FCPA (1977)] makes no exemption for business promotion, so marketing and entertainment (if the intention is corrupt) fall within the scope of the Act regardless of value.

## Assessing Governance

### Accountability

The increase in the role of the media (especially through investigative journalism) has fuelled strong public demand for ISGB accountability to stakeholders, including fans and taxpayers (Ionescu, 2015). Conversely, it has been argued that the media has facilitated corruption through biased positive reporting of unethical sporting behavior (Whannel, 2002, pp. 290–292; Numerato, 2009), such as hailing cheats as national heroes if a country has won a major sporting event despite corrupt behavior.

Transparency as a concept is broader than accountability (defined below), and relates to clarity over the structure, funding, spending, and conduct of an organization through reporting “rules, plans, processes and actions” (Transparency International, 2017c), although disclosure is an important aspect of transparency, such as that found in the likes of the UK Corporate Governance Code (FRC, 2016). Care, however, must be taken to avoid an “accountability-by-audit approach” (where transparency in process is merely a means to allow audit) (Henne, 2015), where generalist rules are not necessarily suitable for the industry.

Klitgaard (1988) and Rose-Ackerman (1999) correlate greater levels of administrator accountability to lower levels of corruption and, therefore, bribery. In the case of (mainly private company) ISGBs, it follows that, where there are no effective mechanisms to hold senior officers accountable for their actions, there are likely to be higher levels of bribery.

Accountability has been defined as holding organizations “responsible for reporting their activities and executing their powers properly” (Transparency International, 2017b), or having actors hold others to a set of standards with sanctions available if these are breached (Stiglitz, 2003; Grant and Keohane, 2005). This includes clear lines of reporting for members, employees, and other stakeholders being available, usually defined in policies and procedures. Non controls (mainly policy) definitions of accountability focus on actions over liability, although there is confusion over the definition (Mcgrath and Whitty, 2018). Accountability was defined in the controls sense in this paper, as having a set of standards such as reporting on specific tasks, having named senior officers responsible for clearly identified specific tasks, and/or the organization or senior officers being explicitly responsible for particular functions or actions within an organization.

Transparency and accountability contribute to ABC as scrutiny of governance leads to lower levels of bribery. For example, Duggan and Levit (Duggan and Levitt, 2002) found that increased media attention decreased match-fixing in sumo wrestling.

### Monitoring/Control

Monitoring is a form of resource control (Lipicer and Lajh, 2013) and can include the use of compliance functions or ethics audits (Mcnamee and Fleming, 2007).

#### Whistleblowing

One of the key methods of monitoring and control center around whistleblowing. Whistleblowing policies allow members to raise concerns about breaches of ethics, laws, and business standards, and enable monitoring and control. For example, the ACFE (2016) noted that “tips” was the most likely form of detection but that “organizations with reporting hotlines were much more likely to detect fraud through tips” than those without. The importance of whistleblowing is also recognized in Transparency International's (Transparency International, 2013a, Article 6.5;2017a, Article 9.2) ABC guidance, and increasingly by policy (see Sport Whistle, 2018) and sports organizations (Cottrell and Erickson, 2018).

The confidentiality and safety of whistleblowing hotlines is important for encouraging witnesses to come forward with information (Soon and Manning, 2017). This is recognized in various statutes worldwide, although cultural differences pervade. Transparency International's (Transparency International, 2013b, p. 8) review of the whistleblowing laws in the EU found only four countries (Luxembourg, Romania, Slovenia, and the UK) had advanced provisions in their laws for “whistleblowers in the public and/or private sectors,” while seven had none or very limited provisions. The EU stance on whistleblower protection has since been enhanced with the advent of the Directive on the protection of persons reporting on breaches of Union law (European Parliament Council of the European Union, 2019). The Sarbanes-Oxley Act (Congress.Gov, 2002, §1514A) in the US also penalizes retaliation against whistleblowers.

### Enforcement

Even if whistleblowing policies exist, enforcement of witness protection and confidentiality rules and regulations increase the tendency for whistleblowers to come forward with information (ACFE, 2016). This enforcement ability also applies to all aspects of governance policy and procedure, as enforcement is, to a degree, positively correlated with adherence by individuals subject to it (Croall, 2004).

Investigatory and enforcement powers are linked to accountability, as ability to enforce decisions independently signals that those in breach of policies and procedures will be held accountable for doing so. (Geeraert et al., 2014) assessed the corporate governance quality in 35 Olympic sport governing bodies, including enforcement powers of the Ethics/Integrity Committees of their sample ISGBs. Only one (UCI) had the ability to initiate proceedings independently at the time.

From an economics perspective, Becker's (1968) rational choice theory was adapted to model public corruption utility choices in South Korea and Hong Kong (Jin-Wook, 2009). This approach was criticized for its simplicity, and was consequently updated by Nichols (2012) to include the ability to use the bribe in secret, perceived (rather than actual) risks of detection, and emotional and psychological costs of acting corruptly. It therefore helps explain why penalties (both criminal and social) form an important part of ABC strategy, as enforcement powers are needed to impose sanctions.

## Method

The anti-bribery framework developed by Philippou (2019) was used to critically evaluate the publically available ABC policies and procedures of 22 ISGBs (listed in Table 1). Assessing the ABC methods employed requires substantive disclosure evidence from the ISGBs on their internal organizational structures and policies employed. This is not always available, and differs from ISGB to ISGB.

**Table 1:** Sampled sports and ISGBs.

**Sport**	**Governing body/bodies**	**Abbreviation**
Athletics	International Association of Athletics Federations/World Athletics	IAAF
American Football	International Federation of American Football	IFAF
Baseball	World Baseball Softball Confederation	WBSC
Basketball	International Basketball Federation	FIBA
Boxing (professional)	World Boxing Association	WBA
	World Boxing Council	WBC
	International Boxing Federation/USBA	IBF/USBA
	World Boxing Organization	WBO
Cricket	International Cricket Council	ICC
Cycling	Union Cycliste Internationale	UCI
Football	Fédération Internationale de Football Association	FIFA
Formula 1	International Automobile Federation	FIA
Golf	International Golf Federation	IGF
Hockey and ice hockey	International Ice-Hockey Federation	IIHF
	International Hockey Federation	FIH
Horse-racing	International Racing Bureau	IRB
Rugby	World Rugby [Rugby Union]	-
	Rugby League International Federation	RLIF
Skiing/snowboarding	International Ski Federation	FIS
Tennis	International Tennis Federation	ITF
Volleyball	International Volleyball Federation	FIVB
Olympics	International Olympic Committee	IOC

In line with the concept of economic rent influencing corruption (Klitgaard, 1988), the sample of ISGBs used in this research were the largest. This conforms with findings by Maennig (2005), where only sports able to generate high income (and economic rents) were affected by corruption, although these findings may have been affected by selection bias in classifying “major documented cases” (p. 190). This approach is also consistent with the sample selection by Geeraert et al. (2014), and Gorse and Chadwick (2011) in their analyses of governance issues and corruption in sport respectively.

Arriving at a sample of the ISGBs with the largest revenues was hindered by some ISGBs not publishing their financial statements (covered in the transparency and accountability section below). Therefore, the list of profitability by sport is an incomplete one. The researchers proxied size to popularity, as defined by their visibility in the media and on terrestrial broadcasting in the largest sports markets (Chadwick, 2013, p. 515; Geeraert et al., 2014, 2015; PWC, 2016). Table 1 lists the sports sampled and their associated ISGBs. Note that the IOC was included (and referred to as an ISGB) in this paper because compliance with the IOC's regulations are the explicitly stated basis for many other ISGBs' policies.

All available documents on structure, governance, financial controls, integrity, and anti-corruption were downloaded from the sample ISGB websites in May 2017 and December 2020 and content analysis performed thereon. The analysis was performed on two dates to also assess ISGB progress with regards ABC policies.

Of the 22 ISGBs reviewed, one (IRB, 2017, 2020) had no relevant documents available on their website (during either timeframe).

The diversity of information available, and the relevant conclusions drawn from this, is discussed in the section on transparency and accountability.

Thematic analysis was undertaken on the ISGB documents available following the approach set out by Braun and Clarke (2006, 2016), followed by the thematic [based on the Philippou (2019) ABC framework] evaluation of the qualitative results (Stemler, 2001; Robson and Mccartan, 2016; p. 349). These 95 (in 2017) and additional 82 (in 2020) documents were reviewed and analyzed by the researchers, using NVivo qualitative data analysis software. The first stage of coding involved reviewing the policies within the documents. Themes were generated from an initial coding run to identify any themes related to anti-bribery and corruption. A second stage of coding was then conducted on the content identified, amalgamating any related codes (such as cash and monetary payments) and splitting any codes that required it (such as ABC). The codes were then compared to the framework and a final analysis was performed on the data to ensure both the themes arising from the data and framework concepts were covered in the analysis. The process was then repeated in 2020 with the additional/updated documents downloaded in December 2020.

## Results

Of the 22 ISGBs reviewed, 14 had an ABC policy of some description in place in 2017, while a third had none publicly available. In 2020, this was increased to 17 so that the absence of ABC policies was less common across the group. Eight ISGBs in 2017 and nine in 2020 had specific ABC policies, while others had included them within other documentation such as a Code of Conduct, Code of Ethics, or Constitution. This has implications for all elements of the assessing governance section of the anti-bribery framework, as lack of easy-to-find, clear-cut policies might limit the strength of the internal control system. It also supports the argument for increased need for staff training on the topic.

Another issue was the inconsistency within ISGBs' policies. An example of poor practice in the 2017 batch was the ITF's (a private registered UK company subject to the UK Bribery Act 2010) policy, which noted that “payment of facilitation payments by or on the behalf of the ITF is therefore only permitted if the following conditions are met …” (ITF, 2012). This implied that facilitation payments are acceptable under certain circumstances, although it then contradicts this in the same document by (correctly, for a company registered in the UK) defining facilitation payments as an example of non-permissible bribery (ITF, 2012). In the 2020 sample for coding, the Anti-Bribery and Corruption Code of Conduct had been replaced by two anti-corruption program documents (ITF, 2020a,b), neither of which specifically referenced bribery. Bribery was instead referenced in the general anti-bribery and corruption clause in the ITF Code of Ethics (ITF, 2019).

## Clarifying Concepts—Definitions

References coded to bribery and corruption themes included:

- Specific details on who is subject to the policy/procedure
- Specific anti match-fixing policy
- Definitions of bribery and corruption
- Examples of bribery and corruption

Who was subject to the policies differed across ISGBs sampled. All applied to officials (see, for example, IFAF, 2012; IIHF, 2014a; World Rugby, 2017; Wbsc, n.d.) and/or athletes and their representatives (IIHF, 2014a; WBC, 2015b). Some had a very broad stakeholder scope, including “the cities and countries wishing to organize” competitions' (FIBA, 2014a), or ‘Representatives of sponsors, partners, suppliers, ski industry and media dealing with FIS and/or have an involvement in FIS activities' (FIS, 2016b). These present best practice solutions for corporate governance issues as put forward by Michie and Oughton (2005) and Pielke (2016). Some ISGBs specifically referred to stakeholders as a “family” (FIFA, 2012a; IAAF, 2015), re-enforcing the idea of self-governing autonomy (Forster and Pope, 2004; Forster, 2006), but also potentially contrary to the independence ideals embedded in a culture of accountability and transparency (Geeraert, 2016; Maennig, 2016).

The ITF and FIA were the only two from the 2017 sample of ISGBs that defined the term bribery as “the offering, promising, giving, accepting or soliciting of an advantage (whether financial or otherwise) as an inducement for an action which is illegal or a breach of trust” (ITF, 2012), or the more specific “improperly influenc[ing] anyone, or … reward[ing] anyone for the performance of any function or activity, in order to secure or gain any commercial, contractual, regulatory or personal advantage” (FIA, 2017b). In the 2020 sample, FIFA defined bribery as an “offer of anything valuable with the intent to gain an improper business advantage” (FIFA, 2020e) and the IRL as “an inducement or reward offered or promised in order to gain any commercial or other advantage” (IRL, 2020a). These are in line with the general definitions discussed previously (2002; Transparency International, 2017b). The FIA further illustrate best practice by providing examples, including “the giving of aid or donations, the use of voting rights, designed to exert improper influence” (FIA, 2017b).

Where references existed to bribery, the second round of coding for each batch determined if non-financial bribery was included (which is definitional-dependent). Non-financial bribery is defined in this paper as the exchange of something other than money in the course of the bribe, such as votes, personal or political favors, or role allocation within an organization. Non-financial bribery was defined in one of three ways in the sample, with some ISGBs incorporating more than one definition:

- “benefit or service of any nature” (see, for example, FIBA, 2014a; IIHF, 2014a; IGF, 2016b; FIA, 2017b)
- pecuniary/ monetary or other benefit/advantage (FIFA, 2012b; ITF, 2012; IAAF, 2015; FIS, 2016b)
- “concealed benefit” (see, for example, FIH, 2012, n.d.; IOC, 2015a; UCI, n.d.)

Non-financial bribery aligns with the “breach of trust” element of the Transparency International (2017b) definition of bribery, and aligns with corruption seen in the 2015 FIFA scandal, where favors were allegedly swapped for votes (Conn, 2018).

The importance of reputational risk to ISGBs was noted, including references to “illegal, immoral and unethical behavior” (FIFA, 2012b), “foster[ing] public confidence in … governance and administration” (ICC, 2014a), “refrain[ing] from unethical behavior that may bring disgrace to many people involved in the sport” (WBC, 2015a), and “not act[ing] in a manner likely to tarnish the reputation of the Olympic Movement” (IOC, 2020a). IFAF summaries this as “Public confidence in the authenticity and integrity of the sporting contest and in the uncertainty of its outcome is vital. If that confidence is undermined, the very essence of the sport is compromised” (IFAF, 2017a). These results support the narrative that integrity of sport is important to ISGBs.

It follows that ISGBs should therefore value ABC, given bribery's damaging nature to integrity (Gorse and Chadwick, 2010). In line with this, unactioned bribery should be covered in best practice ABC policies, and it was indeed covered by some ISGBs. For example, reference was made to breaches occurring “irrespective of whether such benefit is in fact given or received” (IGF, 2016a). However, it could be argued that unactioned bribery is covered by the term “bringing the sport into disrepute” (see, for example, IFAF, 2017b). The issue, from an enforcement perspective, is the breadth of the latter term may make it harder for investigators to prove compared to breaches of specifically referenced bribery, and thus best practice should include specifics.

## Assessing Risk Factors

### Culture

#### Gifts and Entertainment

Documents were coded to the “gift and entertainment” theme if they provided guidance for accepting and/or providing gifts and entertainment to other parties.

Part of the difficulties faced by ISGBs is having to balance international compliance requirements against cultural problems (Pitt and Abratt, 1986) that may ensue in, for example, countries where it is considered rude to decline a host's gift or entertainment offers (Steidlmeier, 1999). This has led to some ISGBs providing generalist policies in their ABC efforts, such as “The hospitality shown to the members and staff … and the persons accompanying them shall not exceed the standards prevailing in the host country” (FIBA, 2014a). This “reasonableness test,” whereby an assessment by members is required, suffers from the same self-regulation enforcement problems that ISGBs are facing with regards general governance (Geeraert et al., 2015; Chappelet, 2016). The FIA was the only ISGB in the sample to explicitly state that “the intention behind the gift should always be considered” (FIA, 2017b).

In line with Transparency International's (Transparency International, 2013a) ABC Principles, perception appears to matter to ISGBs. The ICC (2014a) explicitly forbid gifts that “influence or appear to influence the recipient in the discharge of his official duties,” as do FIBA (2015) “in circumstances that the Participant might reasonably have expected could bring him or the sport into disrepute.” The FIA acknowledges the importance of transparency, in line with Nichols (2012), stating that a condition required of gifts is that they are “given openly, not secretly” (FIA, 2017b), while FIFA (in the 2020 sample) “uses a standard process to register gifts and hospitality and expects every FIFA team member to follow it” (FIFA, 2020d).

Other best practice approaches were adopted by ISGBs. ISGBs referenced gifts of a nominal, trivial, and/or symbolic value only as being acceptable (see, for example, FIH, 2012; IIHF, 2014a; FIFA, 2020f; UCI, n.d.), although arguably this also requires a degree of reasonableness to be applied. Some ISGBs explicitly prohibited the giving/receipt of “cash and cash equivalents” (FIFA, 2012b; ITF, 2012; ICC, 2014a; IAAF, 2015; FIA, 2017b; IFAF, 2017b) or “cash in any amount or form” (FIFA, 2020f). Few ISGBs specified amounts above which gifts and entertainment were considered unacceptable (ICC, 2014a,b; IFAF, 2017b). Aside from providing the basis for ABC financial controls, these policies also provide increased accountability for members” actions (Ionescu, 2015; Pielke, 2016).

Specific circumstances are also considered when forming a gifts and entertainment anti-bribery policy. For example, bribery linked to vote-rigging is explicitly considered in relation to IOC presidential elections: “Candidates may in no case and under no pretext give presents, offer donations or gifts or grant advantages of whatever nature” (IOC, 2015a). IFAF considers procurement in its policy noting that “Particular care must be taken in relation to gifts offered by suppliers, other commercial partners and interested parties to influence decisions relating to the awarding of commercial contracts with IFAF, particularly for media rights, events and sponsorship” (IFAF, 2017b).

Finally, consideration of what to do with gifts that have already been accepted is outlined. For example, the IOC policy that gifts ineligible for acceptance “must be passed on to the organization of which the beneficiary is a member” (IOC, 2015a, 2020a) is also found in other ISGBs (FIBA, 2014a; IGF, 2016c; FIA, 2017b). While setting out parameters for accountability (Geeraert et al., 2013; Maennig, 2016; Menary, 2016), this still presents a problem of what should be done subsequent to this. For example, following the Brazilian Football Association's distribution of Parmigiani watches to FIFA officials, the investigatory chamber decided against formal ethics proceedings “should all watches be returned to it. The watches will then be donated to an independent non-profit organization or organizations committed to corporate social responsibility projects in Brazil” (FIFA, 2017a).

## Assessing Governance Factors

### Accountability

#### Governance Aims

Although forming part of the sport industry typology, differences in the ISGBs' aims may explain the lack of consistency in policies and procedures. For example, most ISGBs in the sample included both promoting/developing and setting the laws of their sport in their mission statements or equivalents. All ISGBs in the sample were hierarchical (Morgan, 2002) in their governance, and the ISGBs did indeed present a different approach to corporate governance compared to other charitable or corporate organizations, with a clear industry-specific focus. For example, the Olympic Charter (the statutes of the IOC), that a large number of ISGBs are signatories of, includes “preserv[ing] the autonomy of sport” (IOC, 2015b, 2020b) in its mission.

In other areas, however, this commonality in aims diverges, with some ISGBs having non-standard aims. The ISGB aims not explicitly shared across the sample include “deliver[ing] commercial value” (RLIF, 2017), providing “editorial services to … publications” (IRB, 2017), and “upholding the interests of its members in … tourism” (FIA, 2017c).

Aims are also likely to be influenced by their income sources. For example, the majority of FIFA's 2016 income came from licensing rights to third parties (FIFA, 2017b) compared to World Rugby (2016) from merchandising (directly from fans). This has implications for both conflict of interest (Brooks, 2016; Kirkeby, 2016) and regulatory arbitrage (Pielke, 2016). Both these ISGBs then saw the majority of their income come from broadcasting in 2019 (FIFA, 2020a; World Rugby, 2020b), a change that also has similar implications.

Some ISGBs govern over leagues with sufficient (usually economic) power to provide them with a voice in their own governance. For example, Formula 1 (FIA, 2017c, 2020b) and the NBA (FIBA, 2014b, 2019) have representation on decision-making committees in their relevant sport, as manifested in their statutes which may affect implementation of best practice (either positively or negatively).

In a similar way, the power of certain countries are also manifested in statutes of relevant ISGBs. For example, World Rugby representatives on the Council have a vote specifically allocated to “Unions … who play in … the Six Nations or SANZAR Rugby Championships” (World Rugby, 2017).

Despite this diversity, most ISGBs note the importance of integrity and reputation, supporting Gorse and Chadwick (2010). For example, FIFA and the UCI both aim “to promote integrity, ethics and fair play with a view to preventing all methods or practices, such as corruption, … which might jeopardize the integrity of” the sport (FIFA, 2016; UCI, 2016) and the WBC (2015a) to promote “Clean, Fair, and Equitable Competition.” Thus, this makes the existence of ABC policies both advisable and desirable within their own stated aims.

#### Transparency and Accountability

Transparency is proxied as public availability of information. One of the ISGBs reviewed had no relevant documents available on their website, although they did have some very limited information relating to aims and contacts (IRB, 2017), and so were included in the analysis.

References demonstrating best practice accountability and transparency are set out in Table 2.

**Table 2:** Examples of best practice accountability and transparency policies.

**Best practice accountability and transparency policy demonstrated**	**Example(s)**
To whom ethics or other policy breaches should be reported	“the FIFA Compliance Division” (FIFA, 2020d)
Who appoints the Ethics Committee or Ethics/Integrity Officer	“ICC's Board of Directors” (ICC, 2014a, 2017)
Who the Ethics Committee members and/or Ethics/Integrity Officer(s) are	World Athletics (2020)
Who and/or what department holds information regarding conflicts of interest and/or policy breaches	“the 6 members of the Ethics Committee and the 2 members of the Secretariat of the Ethics Committee only” (FIA, 2017a)
Who the signatories are for high-value expenditure	“the General Secretary or the Deputy General Secretary” (IIHF, 2014b)
What meeting minutes are kept	“the transcript of the debates of the General Assembly and World Councils, which are recorded on tape” (FIA, 2017d, 2019), although the feasibility of access is unclear
Who and/or what department retains meeting minutes	“The Secretary General is responsible for the minutes of the Congress” (FIS, 2016c, 2018) “Minutes shall be taken of every Congress” (UCI, 2016, 2019)
What Committees and/or Commissions exist and what their responsibilities are	World Athletics (2020)
How officers are nominated	“Nominations Committee” FIA (2017d)
Whether accounts are audited and, if so, who appoints the auditor	FIH (2012)
What activity reports are available, to whom, and how copies can be obtained	FIS (2016c, 2018)

Overall, the levels of ISGB accountability were inconsistent both within and across ISGBs, as was the type of accountability demonstrated. For example, the RLIF (2017) did not include any of the above points in the 2017 sample, but did note the need for “communicating openly and transparently.” In the 2020 sample, they noted that “An up-to-date register of interests will be maintained by the IRL” (IRL, 2020b), although the document did not specify individual roles accountable for this maintenance or review of potential conflicts. No single ISGB included information on all the points in Table 2. These findings are consistent with previous studies on ISGB accountability (Chappelet and Mrkonjic, 2013; Geeraert et al., 2013; Geeraert, 2016) and demonstrate the continued need for accountability in best practice ABC.

Some of the ISGBs published the names of the various committee members, often on their websites, or noted that they ‘shall be published' (FIS, 2016b), but were not available on the website in the 2017 or 2020 reviews. ISGBs also noted specific responsibilities attached to roles, such as “the Chief Administrative Officer shall … see that FIA accounts are kept up to date” (FIA, 2017d, 2019). Some ISGBs also noted specific powers attached to roles, such as “the Central Board has the powers … to exercise overall control over the financial management” of FIBA (2014b; 2019). The latter finding showcases the officers' discretionary powers in a transparent way, which is a positive step toward minimizing corruption (Klitgaard, 1998a; Rose-Ackerman, 1999; Jain, 2001).

Some ISGBs made particular reference to accountability and transparency in their documents. Examples include the “basic universal principles of good governance of the Olympic and sports movement, in particular transparency, responsibility and accountability, must be respected” (FIH, 2012), that “all bodies, whether elected or appointed, shall be accountable to the members of the organization and, in certain cases, to their stakeholders” (IOC, 2015a, 2020a), and to “seek transparency and strive to maintain a good compliance culture with checks and balances” (FIFA, 2012a). In the 2020 sample, it was noted that “One of the fundamental pillars of FIFA 2.0 is the transparency of the organization, its governance and the decision-making process” (FIFA, 2019).

### Monitoring/Control

#### Whistleblowing

References to whistleblowing in the sample were scarce in the 2017 sample. The ITF (2012) noted that a policy exists, but as this was only internally available from the “HR department or in [the] HR shared files,” its contents could not be reviewed by the researchers. The WBSC made reference to whistleblowing in case of actual or “probable cause to believe” (Wbsc, n.d.) a breach has occurred, but no system to do so was set out in their documents.

Direct references to reporting hotlines in ISGB documents, something highly important for monitoring (Transparency International, 2013a, 2017a; ACFE, 2016), was also scarce in the 2017 sample. Some referenced their own (IOC, 2015a; IGF, 2016a, 2017a; FIFA, 2017a,c; UCI, 2017), and one asked their members to use the IOC's Integrity and Compliance hotline (FIS, 2016a). Of those with their own hotlines, one related solely to doping (UCI, 2017) and therefore cannot be considered as part of general ABC policy. Only the IOC had a clear and easy-to-find hotline if one followed the documented references. The IGF hotline was unavailable from the link listed in their Anti-betting and Corruption Policy (IGF, 2016a) when the researchers attempted to access the link in both March and December 2017, but was available from a different URL (IGF, 2017b) after a brief search on the IGF website. Difficulties were also experienced with the FIFA hotline. While FIFA documents made reference to a hotline being set up (FIFA, 2017a) and monitored (FIFA, 2017c), the researchers were unable to find a direct link to this from the FIFA website as at both March and December 2017, although they found the link to FIFA's hotline clearly referenced on, and accessible from, the IOC's website (2017). These findings are in line with the whistleblowing shortcomings discussed by Cottrell and Erickson (2018), and the alleged treatment of whistleblowers by FIFA in the 2015 scandal (Conn, 2018).

The 2020 sample shows that there has been some improvement across ISGBs on this front, with integrity hotlines available across a number of ISGBs (ICC, 2020; TIU, 2020; World Rugby, 2020a; see, for example, FIS, 2020). IFAF has a whistleblower policy document available on their corporate documents webpage (IFAF, 2017c), although there was no other reference to this. The IGF has a dedicated hotline section (IGF, 2020a), although the link to their Anti-betting and Corruption Policy (IGF, 2020c) did not work as at December 2020 and there was potential for conflict of interest as ‘The Head of the IGF Integrity Unit is … the person in charge of the IGF Integrity Hotline and is skilled at providing impartial and confidential support to the person reporting” (IGF, 2020b). FIFA resolved their issue for the 2020 sample and had multiple references to their confidential reporting system (FIFA, 2020b), although their Code of Conduct had nine references to report or contact the FIFA Compliance Division but no links to this or how to do this in the document (FIFA, 2020d). World Athletics now has the independent Athletics Integrity Unit's reporting system available (Athletics Integrity Unit, 2020).

There were also limited references to best practice protection of whistleblowers' and/or witnesses' identity in policy breach proceedings to encourage the practice (Soon and Manning, 2017). The IOC (2015a) noted that “A complainant may request that his/her identity not be revealed and that all precautions be taken so that his/her identity is protected,” while the UCI (n.d.) noted that they “shall take all required measures in order to safeguard the interests and personal rights of witnesses and, if necessary, ensure they remain unidentified.” However, the most detailed policy around the anonymity of witnesses was that of FIFA: “When a person's testimony … could endanger his life or put him or his family or close friends in physical danger, the chairman of the competent chamber or his deputy have powers to maintain confidentiality” (FIFA, 2012b).

The ITF (2012) specifically mentioned culture, wishing to “encourage … individuals [to] feel able to raise concerns” and “strictly prohibits the taking of retaliatory action” and in the 2020 sample had set up a new integrity body (TIU, 2020). Other ISGBs with explicit policies on retaliation against whistleblowers (like those stipulated by laws such as Sarbanes-Oxley in the US and the EU Whistleblowing Directive) included the IGF (2017a, 2020d), which “provide protection against any unjustified treatment in the form of providing confidential advice to whistle-blowers… If physical protection is needed, the case is referred to the police” and FIFA (2012b, 2020f).

### Enforcement

This paper's review of enforcement powers of the Ethics/Integrity Committees found that both samples showed very low levels of ISGB Ethics/Integrity Committees with investigatory and disciplinary powers. While most had the power to request information from individuals subject to the ISGB rules and regulations, a small minority had the power to instigate their own investigations. One that did was the IAAF Ethics Commission in the 2017 sample which could work on matters that it “of its own initiative considers to be appropriate for it to undertake” (IAAF, 2015). The 2020 sample showed that a number of the ISGBs had set up independent integrity units (Athletics Integrity Unit, 2020; TIU, 2020).

Even fewer ISGBs had the power to sanction, an important element of ABC to encourage compliance (Croall, 2004). For example, the ICC (2014a) “Ethics Officer … submit[ing] his written report to the … Board for its ultimate determination on what action, if any, should be taken in respect of the alleged violation” takes the power away from the investigator and puts it into the hands of non-independent officers. This was replaced in the 2020 sample with “the Ethics Officer will refer the matter to the Ethics Disciplinary Committee, which shall normally be comprised of the Chief Executive, the ICC Chairman and the Chair of the Audit Committee” unless it “decides that a greater sanction than a warning and/or reprimand is warranted,” in which case it “shall refer the matter to the Ethics Tribunal” (ICC, 2017). Similarly, the FIBA Ethics Council should “submit to the FIBA Central Board a report … noting any breaches of its rules … [and] will propose … sanctions which might be taken against those responsible” (FIBA, 2014a), but not impose those sanctions itself.

Committees” independence (to enable accountability and limit abuse of powers) was also low. However, definitions of independence were not clarified which, given the extent of conflict of interest issues found in sport as highlighted in this paper, should be treated with caution. Sometimes independence is implied but not explicit, such as for “The FIS Ethics Commission [which] is composed of five persons appointed by the FIS Council; three/four external to FIS and one/two members of the FIS Council” (FIS, 2016b). This also links in with the idea of discretionary powers (Klitgaard, 1988).

Enforcement powers for decisions were rare. Instead, many of the ISGB Ethics Committees had the remit to investigate but not sanction, such as in the case of the FIA (2017b, 2020a), where the Ethics Committee “shall submit a report to the President … who may decide to take further action.” Inability to sanction limits the value of the policies (Croall, 2004).

Sometimes there was no clear enforcer defined, which is also problematic from an accountability viewpoint. For example, FIFA (2012b) Code of Ethics notes that commissions “are forbidden … unless the applicable body has expressly permitted them to do so'. The applicable body in question is not defined in this case, nor is an individual or official role named as the decision-maker. This restricts decision-making ability (and therefore accountability), but also arguably provides officials within FIFA and member associations with discretionary powers, something also linked to increased bribery (Klitgaard, 1988).

Some ISGBs publically list violations or decisions. For example, FIBA (2015) “maintains a list of violations and sanctions which is made available on the FIBA website,” while FIFA (2017a, 2020c) publishes Ethics Committee matters and sanctions. This is a positive step toward transparency (Maennig, 2005; Geeraert et al., 2013).

## Progress

There was evident progress in the amount and quality of ABC material provided by ISGBs between the two sample periods (see Table 3), FIFA and the ITF in particular. Aside from illustrating some ISGBs' growing commitment to transparency and accountability, this also supports Ionescu (2015) research on the role of the media in increasing accountability, as these changes were likely brought in as a result of media scrutiny (Ingle, 2017).

**Table 3:** Key policy improvements 2017–2020.

**Framework element**	**Key best practice policy improvements 2020**
Clarifying concepts	More ISGB examples of clear definitions of bribery (FIFA, 2020e; IRL, 2020a)
Culture	Gift registers (FIFA, 2020d)
Accountability	Register of interests (IRL, 2020b) Transparency as a fundamental aim (FIFA, 2019)
Whistleblowing	Improved availability of integrity hotlines (FIS, 2020; ICC, 2020; TIU, 2020) More references across ISGBs to confidential hotlines (Athletics Integrity Unit, 2020; FIFA, 2020d)
Enforcement	Independent integrity units (Athletics Integrity Unit, 2020; TIU, 2020) Sanctioning decisions published (FIFA, 2020c)

## Conclusion

This paper critically reviewed governance policies for the prevention of bribery in a sample of 22 ISGBs using the anti-bribery framework developed by Philippou (2019). The diversity of both the quantity and quality of information on corporate governance and/or ABC policies is part of the problem that needs to be addressed by future guidance. It is difficult for members and stakeholders to know where to look for ABC information, as this is distributed among statutes, codes of ethics, codes of conduct, or other documents. Future quantitative research can be undertaken to assess policy frequency and distribution.

Qualitative examples of both good and poor practice currently followed by some ISGBs were highlighted across both periods and are summarized in Table 4. There was no single area of the framework that ISGBs performed well at as a collective, and there was no single ISGB whose ABC policies were strong across all areas. A recommended subject for further research is whether particular characteristics of ISGBs positively affect particular aspects of their governance and ABC procedures.

**Table 4:** Examples of good practice.

**Framework aspect**	**Examples of good practice**
Definitions	Bribery and corruption clearly defined (FIA; FIFA; IRL; ITF)
	Non-financial bribery covered in discussions of ABC (FIBA; FIFA; FIH; FIS; IGF; IIHF; IOC; ITF; UCI)
	Bribery and corruption examples provided (FIA)
Gifts and entertainment	Clear gift policy with references to: • Influencing actions (ICC; FIBA)
	• Transparency (FIA)	
	• Registers (FIFA)
	• Nominal/symbolic/trivial value (FIFA; FIH; IIHF; UCI)
	• Specific maximum currency value (ICC; IFAF)	
	Cash or cash equivalents prohibited (FIA; FIFA; IAAF; ICC: IFAF; ITF)	
Transparency and accountability	Internal reporting—breaches and responsible individuals (FIA; FIBA; FIFA)
	Ethics Committee or Ethics/Integrity appointments and members (ICC, World Athletics)
	Information gatekeepers (FIA)
	Signatories are for high-value expenditure (IIHF)
	Meeting minutes – keepers and responsible parties (FIA; FIS; UCI)
Whistleblowing	Accessible ABC reporting hotline (FIFA; FIS; ICC; ITF; UCI; World Rugby)
	Confidentiality (FIFA; IOC; UCI)
	Open culture (IGF; ITF; World Athletics)
Enforcement	Independent integrity units (ITF; World Athletics)
	Transparent enforcement decisions (FIBA; FIFA)

There are limitations of using publically available information for this study, as the information may be incomplete. However, this also reflects the lack of transparency and accountability of the ISGBs in question, and arguably contributes to the likelihood of bribery by those charged with governance (Klitgaard, 1988) of sport.

In terms of clarifying concepts (Philippou, 2019), clear ABC policies on their websites, defining bribery, or including unactioned bribery (which would affect reputation), were few, and there were a number of inconsistencies within ISGBs' own policies. Thus, ISGBs should focus on clarity and consistency when strengthening their ABC policies, starting with defining what it is that they expect their members and stakeholders to avoid.

Governance structures found supported the applicability of corporate governance ABC policies to ISGBs, in line with Smith and Stewart (2010) paper on the sport industry's declining uniqueness. Governance aims of the sample ISGBs converged with regards the importance of integrity, supporting research on risks arising from a lack of integrity (Gorse and Chadwick, 2010). This shows the importance of industry reform in line with other industries, as opposed to an introverted outlook often adopted by sport organizations.

Accountability was deemed important by ISGBs in their documents, but no single ISGB included full information on roles, conflicts, personnel responsible, and so on, while one ISGB had no documents available at all. This paucity in transparency was in line with findings by Geeraert et al. (2014) and is another focus for IGBS looking to undertake reform of their governance and ABC policies and procedures.

Clear gifts and entertainment policies existed, but only one specified a maximum acceptable level of spend. Given the number of reputation-afflicting scandals linked to gifts and entertainment, and the cultural shift away from these as a method of doing business, focus on these policies would enhance the current ABC provisions in ISGBs.

The majority of monitoring and control (Philippou, 2019) references related to whistleblowing. These were, on the whole, scarce, with some ISGBs making reference to reporting hotlines, which can help identify breaches (ACFE, 2016), and a minority to protection of whistleblowers, which help more come forward (Soon and Manning, 2017).

Enforcement powers were low, thus limiting their effectiveness (Croall, 2004), without committees having the power to sanction, while a lack of independence in ISGBs sampled increases discretionary powers of governing officials and therefore the likelihood of bribery (Klitgaard, 1988).

There are, of course, limitations to generalizing the results of this study to all ISGBs. Each ISGB, as shown in this paper, caters to different stakeholders, has different aims, with different governance structures, and very diverse revenue streams and levels. However, this study also highlights why best practice needs to be tailored to the sport industry as a whole, and why ISGBs should share and act on good practice (such as the examples provided by Interpol (2020) on their bi-weekly bulletins, or ESSA (2017).

Most importantly in terms of the practical application of this research for best practice, the existence of robust ABC policies and procedures still requires adherence to and enforcement of these principles. For example, FIFA came under criticism in 2017 for not renewing the independent Ethics Committee's terms, thereby damaging ongoing internal investigations into corruption (Conn, 2017a,b).

The need therefore remains for sharing best practice, and providing guidance on, ABC policies for ISGBs, via the IOC or external enforcement organizations such as UNODC (2018) or stakeholder pressure groups such as SIGA (2017). Future research should engage with stakeholders and ABC practitioners to create a practical and realistic blueprint for best practice in the sport industry and beyond (Michie and Oughton, 2005; Pedersen, 2016; Pielke, 2016). This could be done through interviews or focus groups with ABC professionals, sport governance officers, legal personnel, and stakeholders to analyze perceptions of corruption and ABC in ISGBs against the Philippou (2019) and/or the Masters (2015) frameworks. This can be complimented with research ranking ISGBs by expanding Geeraert's (2018) system to include ABC, and to benchmark the ISGBs sampled.

There is a need to fill the research gaps that exist in relation to both the incidence of bribery, and the fight to prevent it, including research around policy issues and requirements for robust ABC policies, in order to allow for “sport played and governed under the highest integrity standards, free from any form of unethical, illicit and criminal activity” (Siga, 2017).

## Author Contributions

CP compiled the dataset and conducted the analysis of the data. CP was the senior author of the paper. TH is the second author. All authors contributed to the article and approved the submitted version.

## Conflict of Interest

The authors declare that the research was conducted in the absence of any commercial or financial relationships that could be construed as a potential conflict of interest.
